# Two dopamine D2-like receptor genes from the silkworm (*Bombyx mori*) and their evolutionary history in metazoan

**DOI:** 10.1038/s41598-017-07055-5

**Published:** 2017-07-28

**Authors:** Ping Chen, Peng Chen, Tian Li, Qi Shen, Deng-Feng Yan, Liang Zhang, Xi Chen, Yan Li, Wei Zhao

**Affiliations:** College of Biotechnology, Southwest University, Chongqing, 400715 China

## Abstract

Dopamine is widely distributed in metazoans and is implicated in many physiological functions. Dopaminergic signaling is mediated through two classes of dopamine receptors, D1-like and D2-like. Phylogeny analysis reveals that, the dopamine receptors probably appeared ahead of the cnidarian divergence, two distinct classes of dopamine receptors likely formed prior to the separation of deuterostomes and protostomes, and INDRs probably split from its ancestor before the emergence of nematodes. Two D2-like genes are closely linked on the same scaffold, and the chromosome region around D2-like gene loci show colinearity among different species within Lepidoptera. These indicate two D2-like and their adjunction genes are likely Lepidoptera-specific orthologs, and occur by gene duplication event taken place after Lepidoptera ancestor split from the common ancestor of Lepidoptera and Diptera. In silkworm, two D2-like genes were expressed in examined tissues, and encoded BmDop2R2 having all the features of D2-like receptors and BmDop2R1 being a truncated variant without the region of N-terminal to TM II. Only dopamine distinctly lowered cAMP levels in BmDop2R2-expressing cells, whereas all tested amines for BmDop2R1 had not markedly effect in pharmacological test. These suggest there is functional difference between the two genes, which are likely resulted from subfunctionalization of gene duplication.

## Introduction

Dopamine is widely distributed in metazoans and is implicated in many physiological functions^1–3^. Dopaminergic signaling is mediated through the rhodopsin-like family of G protein-coupled receptors (GPCRs) superfamily that plays a fundamental role in regulating various cellular activities. GPCRs contain seven trans-membrane (TM) spanning domains with ligand-binding sites, an extracellular amino-terminus, and an intracellular carboxyl tail^4, 5^. In mammals, the dopamine receptor family is divided into two classes (D1-like and D2-like) based on their pharmacological properties and intracellular signaling pathways. D1-like receptors including D1 (D1A) and D5 (D1B) receptors, are coupled to the Gs/Golf class of G_α_ proteins and thereby activate adenylyl cyclase to increase intracellular cAMP levels. However, D2-like receptors including D2, D3, and D4 are coupled to G_α_i/Go proteins, which inhibit adenylyl cyclase thus decreasing the intracellular cAMP levels. D1-like receptors are encoded by intron less genes and exhibit a short third cytoplasmic loop and a long C-terminal tail; whereas D2-like receptor genes contain several introns and encode a long third cytoplasmic loop and a short C-terminal tail^6–11^.

Non-mammalian dopaminergic GPCRs are also subdivided into two major groups (D1-like and D2-like) based on sequence homology, signal transduction mechanisms, and sensitivity to class specific drugs^6, 12^. Further, invertebrate (honey bee, fly and nematode) dopamine receptors are divided into three distinct groups, DOP1, DOP2, and DOP3 (corresponding to Dop1R1, Dop1R2, and Dop2 in this study) in comparison with vertebrates (human and frog)^12^. Like the mammalian D1-like receptors, DOP1 (Dop1R1) and DOP2 (Dop1R2) upregulate cAMP levels when stimulated with dopamine and therefore function like D1-like receptors. Their sequence structure also has relatively short third cytoplasmic loops and relatively long carboxyl tails. But unlike the human D1-like receptors, the genes encoding members of the invertebrate D1-like contain introns^13–19^. The invertebrate Dop2 has a longer third cytoplasmic loop and a shorter C-terminal end which are similar to the mammalian D2-like receptors. Dop2 reduces intracellular cAMP levels in the presence of dopamine, and therefore, functions like D2-like receptors^20–23^.

Since the 1990s, a number of dopamine receptor genes have been identified and isolated from insects including *Drosophila melanogaster* ^14, 18, 21^, *Apis mellifera* ^20^, *Periplaneta Americana* ^19^, *Ctenocephalides felis* ^15^, *Papilio xuthus* ^17^, *Tribolium castaneum* ^23^, *Chilo suppressalis* ^24, 25^ and *Bombyx mori* ^26, 27^. Interestingly, DmDopEcr, a GPCR receptor activated by steroid ecdysone, can be activated by dopamine to increase cAMP levels^28^. In honey bee, AmDop2 is widely expressed in the brain but differs from AmDop1R1 and AmDop1R2 in its expression pattern^20^. In red flour beetle, TcDop2 is highly expressed in both the central brain and the optic lobes, which is consistent with the role of dopamine as neurotransmitter^23^. In schistosomes (*Schistosoma mansoni*), SmDop2 is detected in the sub-integumental somatic musculature and acetabulum of larvae, and in the somatic muscles and the muscular lining of the adult caecum^29^. In flies, DmDop2 is expressed in the larval and adult nervous system, and is shown to regulate locomotion^30^. In Lepidoptera, CsDop2 gene in rice stem borer is identified and pharmacologically characterized in the recent report ^25^, but the knowledge about D2-like receptors is still lacking. In this study, we cloned D2-like receptor genes from the silkworm, *Bombyx mori*, a Lepidopteran model, analyzed their evolutionary relationship to metazoan homologs, and identified their function in human embryonic kidney (HEK) 293 cells.

## Results

### Cloning BmDop2R1 and BmDop2R2

**Figure 1:**
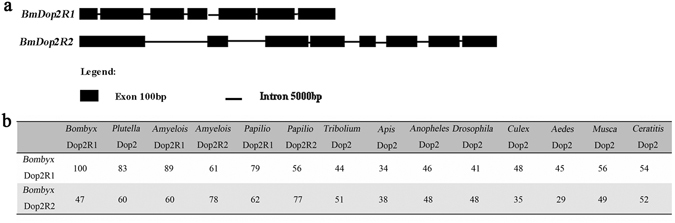
(**a**) The structures of *BmDop2R1* and *BmDop2R2* genes. Exons are represented by black boxes and the intron length is represented by lines. (**b**) Amino acid homology scores among insect Dopamine D2-like receptors. GenBank accession numbers of the proteins used are listed in Supplementary Table S1.

Comparing the amino acid homologous sequences of dopamine D2-like receptors from *D. melanogaster*, *A. mellifera*, *S. mansoni* and *Caenorhabditis elegans*, we searched the database and identified two genes in the silkworm genome. Abiding by the nomenclature of D1-like subclass of arthropod (Dop1R1 and Dop1R2), in this report, one gene having higher homology with the gene of D2-like receptor of other animal was designated as *BmDop2R1*, while the other was designated as *BmDop2R2*. Then, by RT-PCR and the RACE technique we cloned the 1406 bp long BmDop2R1 cDNA sequence containing a 1323 bp long ORF that encoded a protein with 440 amino acids and a predicted molecular mass of 46.29 kDa. The BmDop2R1 gene had seven exons and six introns (Fig. 1a, Supplementary Fig. S1). The cloned cDNA sequence of *BmDop2R2* was 1636 bp long and contained a 1596 bp ORF that encoded a protein with 536 amino acids with a predicted molecular mass of 59.67 kDa. The BmDop2R2 gene contained eight exons and seven introns (Fig. 1a, Supplementary Fig. S1). Using the online domain prediction at EMBL, we found a G-protein coupled receptors family 1 profile in both BmDop2R1 and BmDop2R2 (Supplementary Fig. S2).

### Sequence Analysis

Using the BLAST tool at NCBI we searched the homologs of D2-like receptor genes in the genome databases of insects, and found two D2-like receptor genes in *Plutella xylostella* (Lepidoptera, Plutellidae), *Amyelois transitella* (Lepidoptera, Pyralidae) and *Papilio machao*n (Lepidoptera, Papilionidae), but only one D2-like receptor gene in other species including *A. mellifera* (Hymenoptera), *Tribolium castaneum* (Coleoptera), *D. melanogaster* (Diptera, Drosophilidae), *Culex quinquefasciatus* (Diptera, Culicidae), *Aedes aegypti* (Diptera, Culicidae), *Anopheles gambiae* (Diptera, Culicidae), *Musca domestica* (Diptera, Muscidae), and *Ceratitis capitata* (Diptera, Tephritidae). Thus, Lepidoptera D2-like receptor had two subclass, Dop2R1 and Dop2R2, corresponding to BmDop2R1 and BmDop2R2, respectively. But the similarity of amino acid sequences within Lepidoptera was higher than that among species (detail in Fig. 1b). Unexpectedly, though the nucleotides of two D2-like genes of *P. xylostella* shared only 83% homology, the amino acid sequences coded by both genes were identical with BmDop2R1. Interestingly, the receptor genes identified in *T. castaneum*, *C. quinquefasciatus*, and *A. aegypti* genomes showed great divergence from BmDop2R1 and BmDop2R2. However, the receptor genes of *A. mellifera* and *A. gambiae* were similar to BmDop2R1 and BmDop2R2 (detail in Fig. 1b). For example, the *A. aegypti* D2-like receptor gene shared 45% amino acid similarity with BmDop2R1 and 29% with BmDop2R2, whereas the *A. gambiae* D2-like receptor gene was 46% and 48% similar to BmDop2R1 and BmDop2R2, respectively (Fig. 1b).

**Figure 2:**
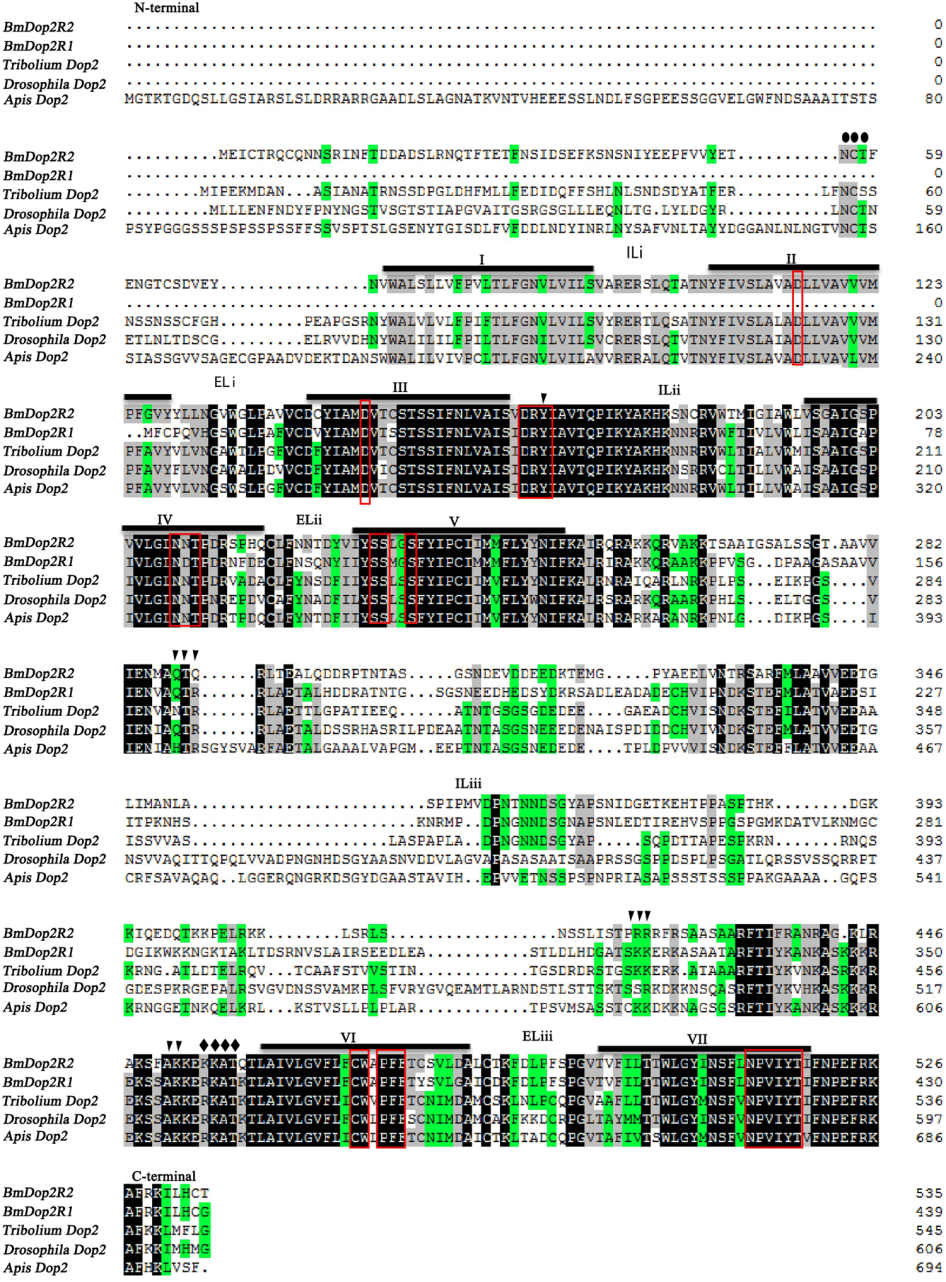
Comparison of amino acid sequences of silkworm dopamine D2-like receptors (BmDop2R1 and BmDop2R2) with *Apis* Dop2, *Drosophila* Dop2 and *Tribolium* Dop2. GenBank accession numbers of the proteins used are shown in Supplementary Table S1. Putative transmembrane domains (TMI-TMVII) are indicated by bold black lines. PKC phosphorylation sites (▼) and potential N-glycosylation sites (●) are indicated. IL represents intracellular loop and EL represents extracellular loop. Red box indicates the ligand-binding site.

Analyses of amino acid sequences revealed that amino acid sequences were conserved mainly in the trans-membrane domains while most differences among species occurred in the amino termini and the non-transmembrane domains (Fig. 2). BmDop2R2 protein contained seven hydrophobic transmembrane domains (TM I-VII) with 3 intracellular loops and 3 extracellular loops (Fig. 2, Supplementary Fig. S2), indicating that BmDop2R2 belonged to the GPCRs family. BmDop2R1 protein had only five hydrophobic trans-membrane domains, and was a truncated variant lacking N-terminal region and the first to second trans-membrane domains (TM I-II) (Fig. 2, Supplementary Fig. S2). However, BmDop2R1 shared structural identity/similarity with other insect D2-like receptors and contained a relatively short cytoplasmic C-terminal tail and a relatively long third intracellular loop (Fig. 2), which were characteristics of invertebrate and mammalian D2-like receptors. In addition, the insect D2-like receptors along with BmDop2R1 and BmDop2R2 also contained several highly conserved sequence motifs and amino acid residues (Fig. 2). For example, the aspartate residue (D) in TM III was predicted to bind the amine group in catecholamines such as dopamine^31^; the serine residues (S) in TM V may form hydrogen bonds with the hydroxyl groups in dopamine^32^; the DRY sequence located at the end of TM III was involved in receptor activation; the characteristic CW-x-PFF in TM VI was critical for interaction with the aromatic ring of dopamine; and the conserved NPVIY in TM VII was crucial to stabilize the inactive conformation of the receptor^33, 34^. Moreover, both BmDop2R1 and BmDop2R2 had a number of potential motifs for protein kinase C (PKC) phosphorylation sites and N-glycosylation sites (Fig. 2). These findings suggested that *BmDop2R1* and *BmDop2R2* encoded *B. mori* dopamine receptor and support the notion that BmDop2R1 and BmDop2R2 were within the biogenic amine receptors superfamily.

### Phylogenetic Analysis

**Figure 3:**
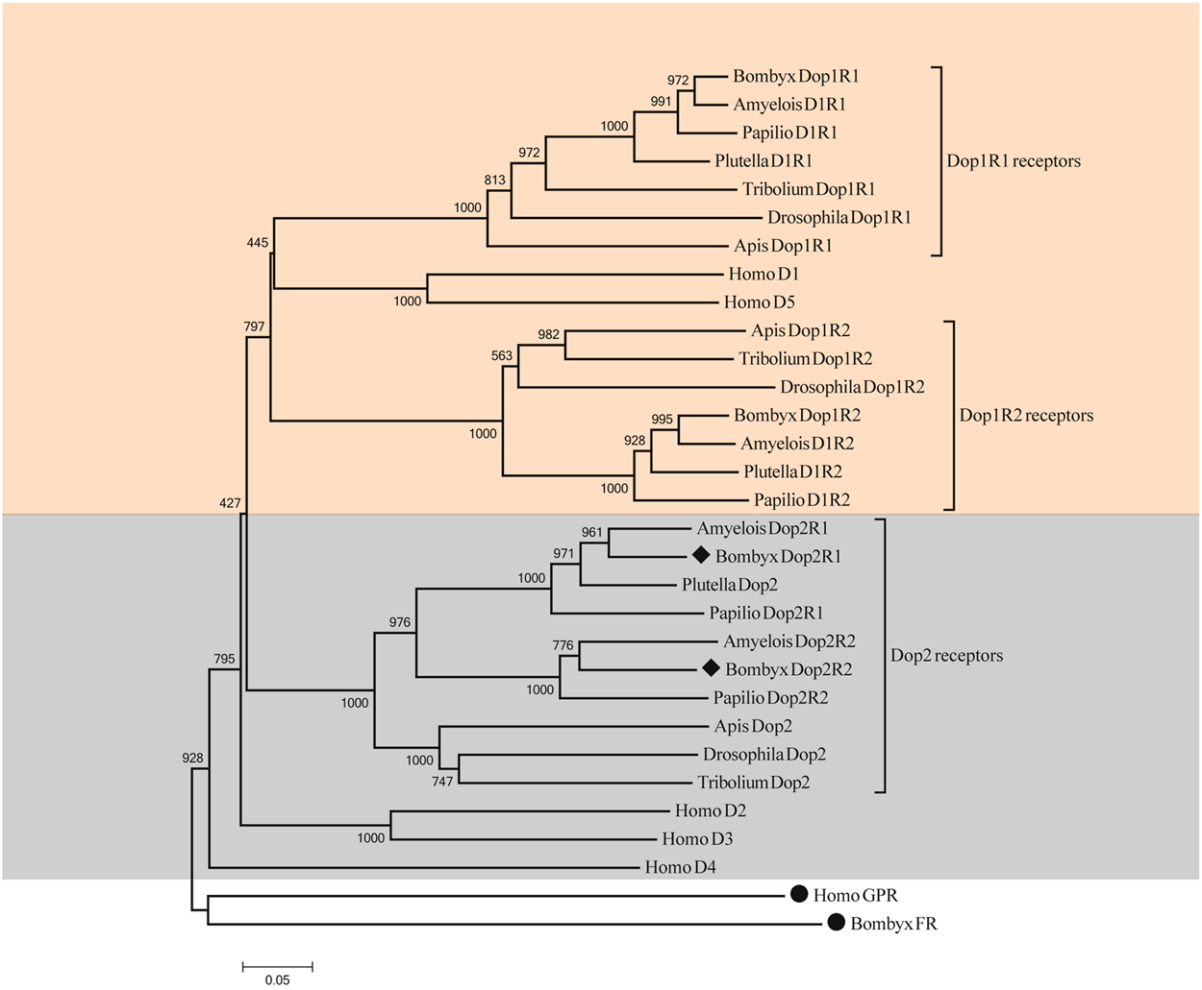
Phylogenetic tree of insect and human dopamine receptors. Human GPR proteins and silkworm FR proteins are used as outgroups. GenBank accession numbers of the proteins used for this phylogenetic analysis are listed in Supplementary Table S1. Dopamine D1-like receptors are indicated in orange and D2-like receptors are indicated in gray.

We constructed a phylogenetic tree of dopamine receptors based on homologous protein sequences from insects and humans (Fig. 3). The out-groups were human GPR54 protein and the silkworm FR protein belonging to the GPCRs family. All dopamine receptors were categorized into four clades. The first clade was D1-like clade, where the insect D1-like divided into two distinct subclades, Dop1R1 subclade and Dop1R2 subclade, and human D1-like (D1 and D5) appears to be closer to Dop1R1 than Dop1R2. Since Dop1R2 was unique to invertebrates it was named as invertebrate-type dopamine receptor (INDR) in the report by Mustard.J A^12^. All insect D2-like clustered into the second clade containing two subclades, Lepidoptera D2-like subclade divided into two distinguishable  branchs (Dop2R1 branch and Dop2R2 branch) and other species D2-like subclade. Silkworm clustered with *A. transitella* not only in Dop2R1 branch but in Dop2R2 branch. Unexpectedly, the human D2 and D3 were incorporated into the third clade, and only the human D4 formed a independence clade.

A BLAST search (http://www.ncbi.nlm.nih.gov/Taxonomy) showed no homologous dopamine receptor gene in the genome of *Amphimedon queenslandica*, an old multi-cellular animal. The genome of *Hydra vulgaris* contains seven sequences with homology to dopamine receptors and includes D(1 A)-, D(1C)-, D(2)A-, D(2)-, and 4-like receptors in annotation. In sea pansy, all three conventional transmitters dopamine, noradrenaline, and adrenaline were identified^35, 36^. In *S. mansoni*, a dopamine D2-like receptor shared high homology with the dopamine receptor prototypes of mammals^27^. It also contains two putative biogenic amine (dopamine, Dop-1 and Dop-2) receptors. Besides, the flatworms clearly have dopamine D1-like and D2-like receptors as evidenced by pharmacological analyses^37, 38^. These suggest that biogenic amine (such as dopamine) receptors likely appeared before cnidarian divergence and that the D1-like or D2-like divergence from the ancestor took place before the separation of platyhelminthes. In *C.elegan*s, there are six dopamine receptor genes including Dop-1 to -6 among which four were isolated and identified as D1-like or D2-like receptors^27, 39–41^. And there are four (three putative and one isolated genes) dopamine receptor sequences from its genome in the mollusk, *Aplysia californic*a. Further, in *Panulirus interruptus* (Arthropoda, Malacostraca)^42^, as well as in *Ixodes scapularis* (Arthropoda, Arachnida)^43^ and only three dopamine receptor genes (two D1-like and one D2-like) were identified in insect. Unexpectedly, we found two D2-like genes from the silkworm or other Lepidopteran genomes. This indicated that the numbers of dopamine receptor genes changed during the evolution of protostome. In parallel, these changes also likely occurred during the vertebrate evolution. For example, two were reported/identified in *Lampetra fluviatilis* (agnathans, jawless vertebrates), thirteen in *Danio rerio* (jawed vertebrates), seven in *Alligator sinensis*, six in *Gallus gallus*, and five in mammals^6, 44^. Such increasing in the number of dopamine receptor genes likely resulted from gene duplication events followed by the selection of these duplicated genes (gene silencing).

**Figure 4:**
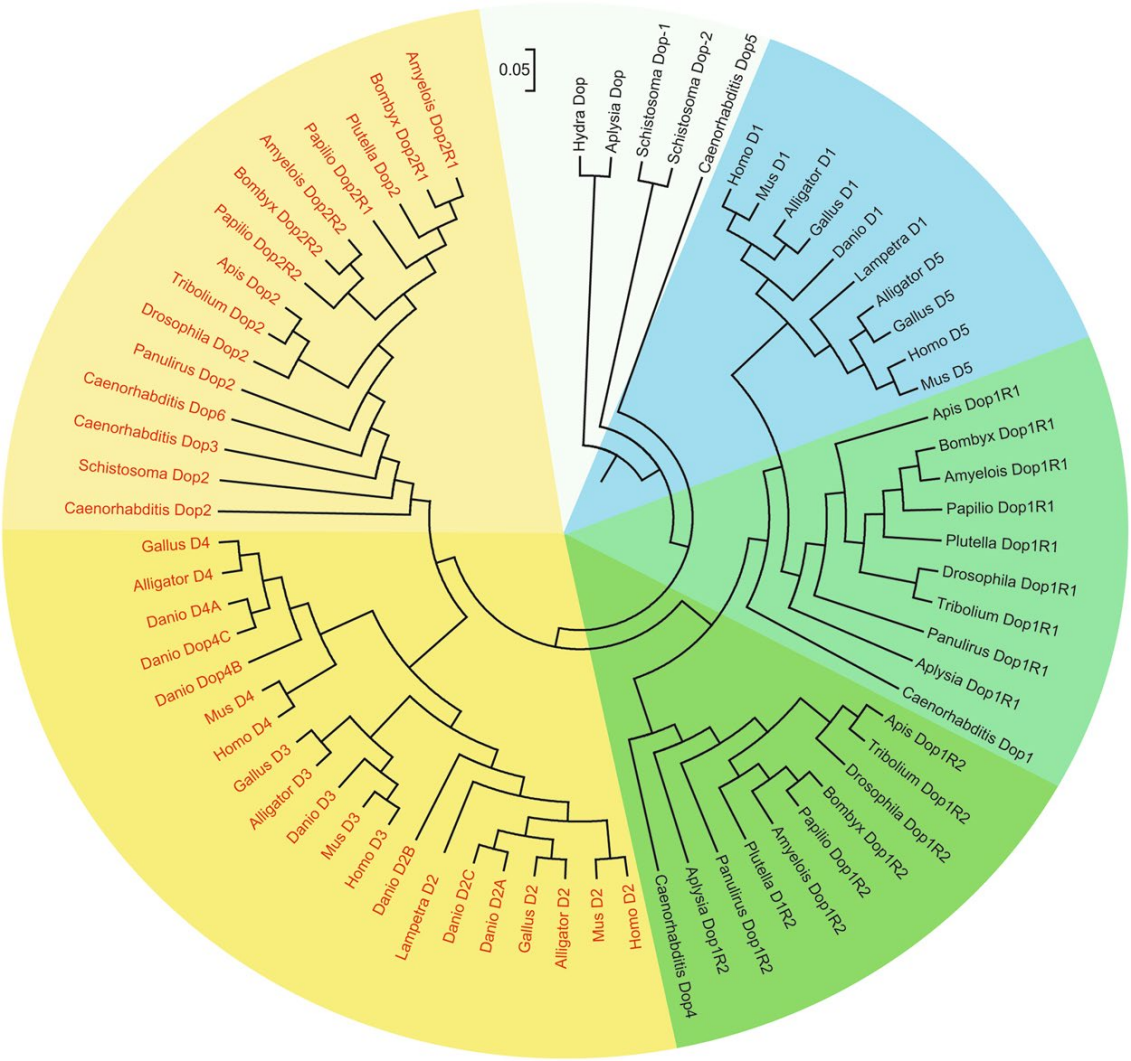
Phylogenetic tree of dopamine receptors from metazoa. GenBank accession numbers of the proteins used for this phylogenetic analysis are listed in Supplementary Table S1. Vertebrate D2-like receptor, D2 as well as D3 or D4 are indicated in yellow. Invertebrate Dop2 (D2-like receptor) are indicated in pale yellow. Invertebrate Dop1R1 are illustrated in pale green and Dop1R2 in green. D1 and D5 of vertebrate D1-like receptors loci in the blue areas. Predicted biogenic amine (dopamine) receptors from low animal as tree root are covered with pale white.

To provide additional evidence for the evolution of dopamine receptors, we used 70 genes from 18 species across metazoa and constructed a second phylogenetic tree (Fig. 4). Since D1 (D1A) and D5 (D1B) exist in all vertebrates, they were used in this tree as a represent for vertebrate D1-like receptors. The *C. elegans* Dop5 was located at the root of the phylogenetic tree comprising two discrete clades, D1-like clade and D2-like clade (Fig. 4). The D1-like clade contained two distinct subclades. The invertebrate Dop1R2s were clustered in the first subclade with INDRs and *C. elegans* Dop4 (identified as D1-like receptor unique to invertebrates^22^). Together with previous work^12–16, 19, 20, 22, 42, 43^, the results suggested that Dop1R2 probably as INDR splits from its ancestor before the emergence of nematodes during the protostome evolution. The *C. elegans* Dop1 (identified as D1-like receptor^40^) as an outgroup was located at the second subclade containing the invertebrate Dop1R1 group (where invertebrate being consistent with the evolutionary relationship were incorporated) and the vertebrate D1-like group (where D1 and D5 were clustered respectively into two individual branches). The D2-like clade contained the vertebrate D2-like subclades and the invertebrate D2-like subclades. The *C. elegans* Dop2, -6 (identified as D2-like receptor^22^), -3 (identified as D2-like receptor^41^) and *S. mansoni* Dop2 (identified as D2-like receptor^27^) were located at the outgroup of the invertebrate D2-like subclades. Lepidoptera D2-like were categorized into two evident branches, Dop2R1 branch and Dop2R2 branch, where the silkworm and *A. transitella* were clustered. This was alike to the manner of previous tree, implying that two subclasses of D2-like likely occurred before species divergence from their common ancestor in Lepidoptera. The vertebrate D2-like subclade comprised of two visible groups, the first group contained the vertebrate D2 branch and the vertebrate D3 branch, and the vertebrate D4 sequences appeared as an independent branches and formed a second group (Fig. 4), suggesting that vertebrate D2 and D3 could have a common ancestor who may not be the recent ancestor of vertebrate D4. Overall, the topologies of the phylogenetic tree were high similar to those in the previously constructed tree. This phylogenetic tree was rooted on putative biogenic amine (dopamine) receptor sequences from lower animals such as *H. vulgaris* [Dop, annotated as D(1 A) dopamine receptor-like], *S. mansoni* [Dop-1 and -2. As putative dopamine receptor sequences, Dop-1 was highly identical degree with histamine receptor-like sequences confirmed by pharmacology experiment^44^], and *A. californica* (Dop) (Fig. 4), implying that these sequences were perhaps related to the prototype biogenic amine receptors such as the dopamine receptor.

### Synteny Analysis

**Figure 5:**
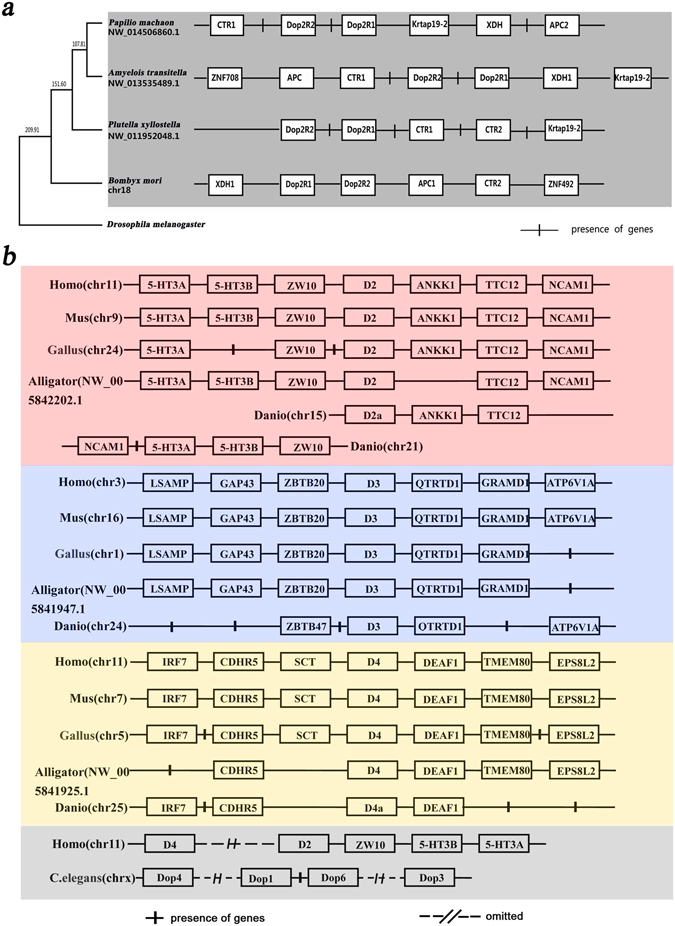
Synteny of D2-like receptor gene loci. (**a**) Synteny of D2-like receptor gene loci in Lepidoptera. The species tree (in the left) constructed by Cytochrome C is illustrated in white. The neighboring genes arounded D2-like receptor gene (in the right) are illustrated in gray. (**b**) Synteny of D2-like receptor gene loci in vertebrates and nematodes. Syntenies of vertebrate *D2* are illustrated in red, *D3* in blue-gray, *D4* in yellow. The linkage of two D2-like genes in human and in a nematode are illustrated in gray.

In this study, we found that the two D2-like genes (BmDop2R1 and BmDop2R2) were located on chromosome 18 even on the same scaffold (nscaf 2901), and there was a microRNA gene between them in the silkworm genome. Interestingly, the close linkage in two D2-like genes was conserved in all the Lepidopteran genomes mentioned above (Fig. 5a). *Dop2R2* located at the downstream of *Dop2R1* in the silkworm but at upstream of *Dop2R1* in others (Fig. 5a). Observing the neighboring genes around D2-like gene loci, there were XDH1 in upstream, APC1, CTR2 and ZNF492 in downstream in the silkworm. And there were CTR1, CTR2 and Krtap19-2 genes in downstream in *P*. *xylostella*; CTR1, APC and ZNF708 in upstream, XDH1 and Krtap19-2 in downstream in *A. transitella*; CTR1 in upstream, Krtap19-2, XDH and APC2 in downstream in *P*. *machao*n genome (Fig. 5a). These indicated that gene colinearity around D2-like loci existed in Lepidoptera. But such synteny did not emerge in non-Lepidopteran species.

Thus, the chromosome region including two D2-like and their adjunction genes were likely Lepidoptera-specific locus where the genes were likely Lepidoptera-specific orthologs. This view was also supported by the above phylogenetic analysis.

By screening the homologs of D2-like genes, we investigated whether the neighboring genes had the tendency to retain their relative positions and orders on chromosomes in the vertebrates *Xenopus laevis* (not show), *Alligator sinensis*, *Gallus gallus*, *Mus musculus*, and *Homo sapiens*. These vertebrates clearly have three subclasses (D2, D3, and D4) in the D2-like class. The results showed a striking conservation in gene synteny in the chromosome region around the D2 gene, as well as around D3 or D4 (Fig. 5b). In *Danio rerio*, there were three subtypes of D2 (D2a, D2b, D2c) as well as D4 (D4a, D4b, D4c), but only one D3. Each subtype was distributed on different chromosomes, and one of the paralogous loci (such D2a and D4a) shared syntenic genes with amniotes, but not others in each subtype. This suggested that the chromosome duplication of a gene was accompanied by a corresponding major rearrangement of one of the duplicated locus. Interestingly, homologous genes located upstream and/or downstream of D2 in amniotes were distributed respectively on chromosome 15 and chromosome 21 in *D. rerio* (Fig. 5b), but were located one scaffold (NW-005819020.1) in *Latimeria chalumnae* (coelacanth, a slightly higher animal than *D. rerio*). This indicated that the genes linked to D2 and their orders in current amniotes likely formed before the amphibian split. In addition, D2 and D4 were linked in series on the same chromosome in only primates such as Human, but not in other vertebrates. This was likely caused by chromosome segment fusion prior to the divergence of primates.

The conserved degree of synteny around D2-like gene was lower among Lepidopteran insects than among vertebrates. It was clear that rearrangements occur more frequently in small mammals with short generation time compared to larger mammals with long generation time^22^. The genes surrounding D2-like receptor genes may change due to gene rearrangements after species divergence from a common ancestor particularly in insects with shorter generation time comparing to small mammals.

### BmDop2R1 and BmDop2R2 expression patterns

**Figure 6:**
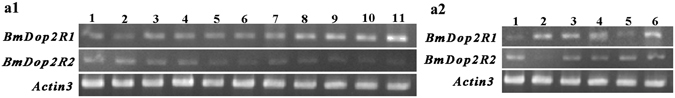
Expression profile of *BmDop2R1* and *BmDop2R2* in larval (**a1**) and adult (**a2**) tissues of silkworm. **a1** - Lane 1: Head; 2: Fat body; 3: Silk gland; 4: Tracheae; 5: Ventral chain; 6: Hemolymph; 7: Testis; 8: Ovary; 9: Integument; 10: Malpighian tubule; 11: Midgut. **a2** - Lane 1: Head; 2: Thorax; 3: Abdomen; 4: Ovary; 5: Testis; 6: Fat body. *BmActin3* was used as the internal control.

We determined the tissue-specific expression pattern of *BmDop2R1* and *BmDop2R2* using RT-PCR. Eleven larval tissues (head, fat body, silk gland, tracheae, ventral chain, hemolymph, testis, ovary, integument, malpighian tubule, midgut) and six adults tissues (head, thorax, abdomen, ovary, testis, fat body) were used for the analysis. *BmDop2R1* and *BmDop2R2* were almost ubiquitously expressed in all tissues examined although with differences in the expression levels (Fig. 6a1). For *BmDop2R1*, an intense product that was 753 bp was detected in the head, fat body, silk gland, tracheae, ventral chain, hemolymph, testis, ovary, integument, malpighian tubule, midgut of larvae, and the thorax, abdomen, ovary and fat body of adults. A relatively weaker *BmDop2R1* product was detected in the larval fat body, and adult head and testis (Fig. 6a1,a2). Expression of the 660 bp *BmDop2R2* product was robust in the head, fat body, silk gland, tracheae of larvae, and the head, abdomen, ovary, testis, and fat body of adults. A weaker *BmDop2R2* product was detected in the ventral chain, hemolymph, testis, ovary, integument, malpighian tubule, midgut of larvae, and adult thorax (Fig. 6a1,a2). These results indicated the functions of both *BmDop2R1* and *BmDop2R2* in the different tissues.

### Functional Analysis

**Figure 7:**
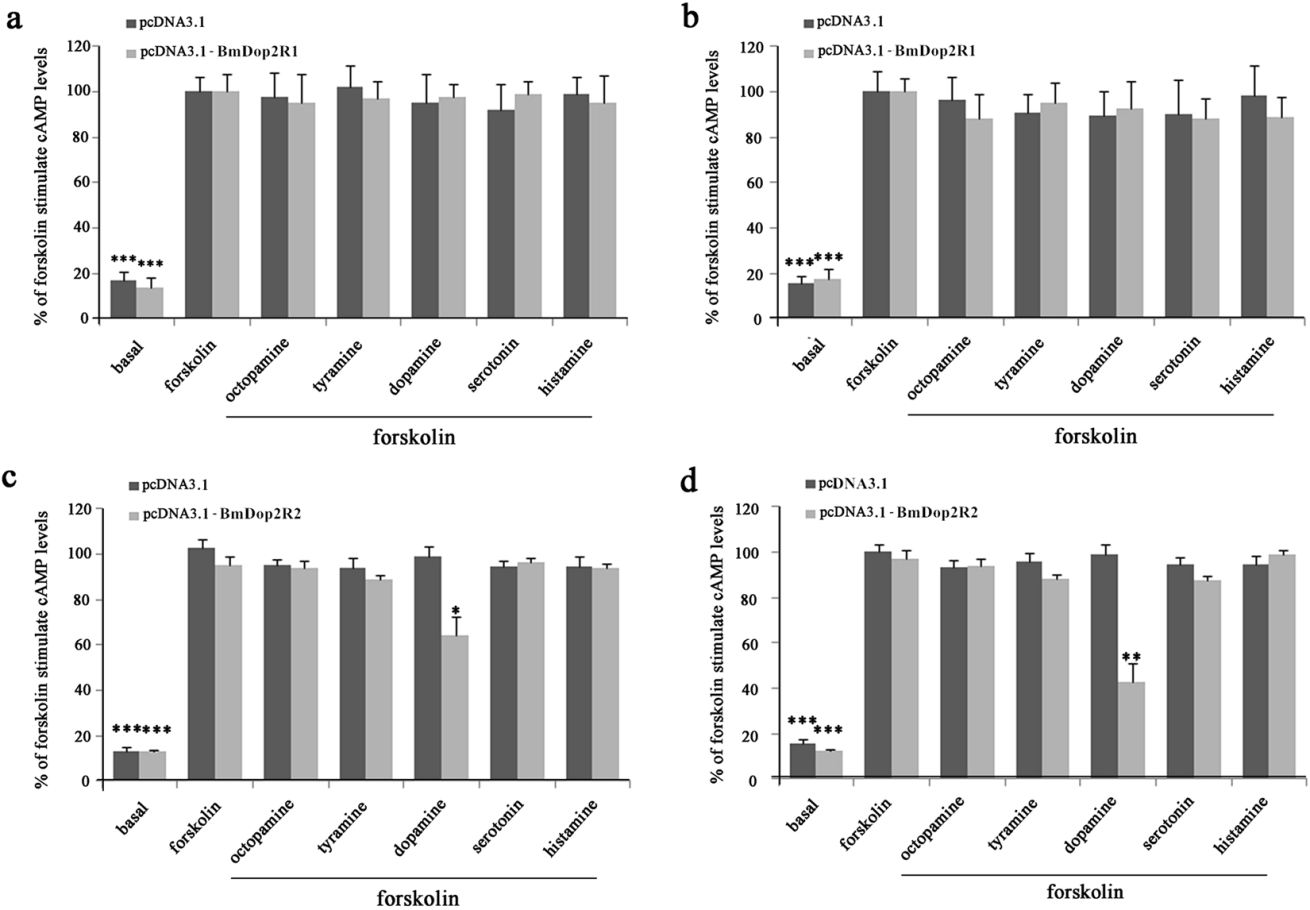
Relative levels of intracellular cAMP in HEK293 cells expressing BmDop2R1 or BmDop2R2 receptor compared to control (empty vector). The 100% level represents the cAMP amount in control cells treated with 10 μM forskolin, and is compared in statistical analysis. Basal represents relative levels of cAMP in untreated cells expressing BmDop2R1 or BmDop2R2 receptor compared to control. (**a**) 1 μM biogenic amines with 10 μM forskolin treating cells expressing BmDop2R1. (**b**) 10 μM biogenic amines with 10 μM forskolin treating cells expressing BmDop2R1. (**c**) 1 μM biogenic amines with 10 μM forskolin treating cells expressing BmDop2R2. (**d**) 10 μM biogenic amines with 10 μM forskolin treating cells expressing BmDop2R2. Data represent the mean of four to six values, and error bars indicate S.E.M. (*), (**) and (***): P < 0.05, P < 0.01 and P < 0.001, respectively.

In insects, five major amines, serotonin (5-hydroxytryptamine, 5-HT), dopamine, histamine, octopamine and tyramine, are involved in intercellular signalling. To determine the ligand specificity of receptors, the HEK 293 cells expressing BmDop2R1 or BmDop2R2 were treated with five biogenic amines (1 μM or 10 μM) respectively. Control was empty pcDNA3.1 vector under the same conditions, and expression was confirmed by RT-PCR using total RNA extracted from transiently transfected cells (Supplementary Fig. S3). Since D2-like dopamine receptors could decrease cAMP levels which were low in normal cells by inhibiting adenylyl cyclase activity, 10 μM forskolin was applicatied to stimulate adenylyl cyclase to increase cAMP levels (Fig. 7) for observing clearly the effect of BmDop2R1/BmDop2R2 receptors.

No significant difference was observed in the cAMP levels among five biogenic amines in empty vector cells (Fig. 7a-d). In BmDop2R2-expressing cells, only dopamine distinctly lowered cAMP levels, which was down-regulated to 72.27% and 47.12% of the control group at 1 μM and 10 μM dopamine concentrations respectively (Fig. 7c,d). These displayed that dopamine was the most potent of the biogenic amines at activating BmDop2R2 receptors. However, all these amines did not induced a markedly change of the intracellular cAMP in BmDop2R1-expressing cells (Fig. 7a,b), or even high concentrations of dopamine (100 μM) (date not show).

**Figure 8:**
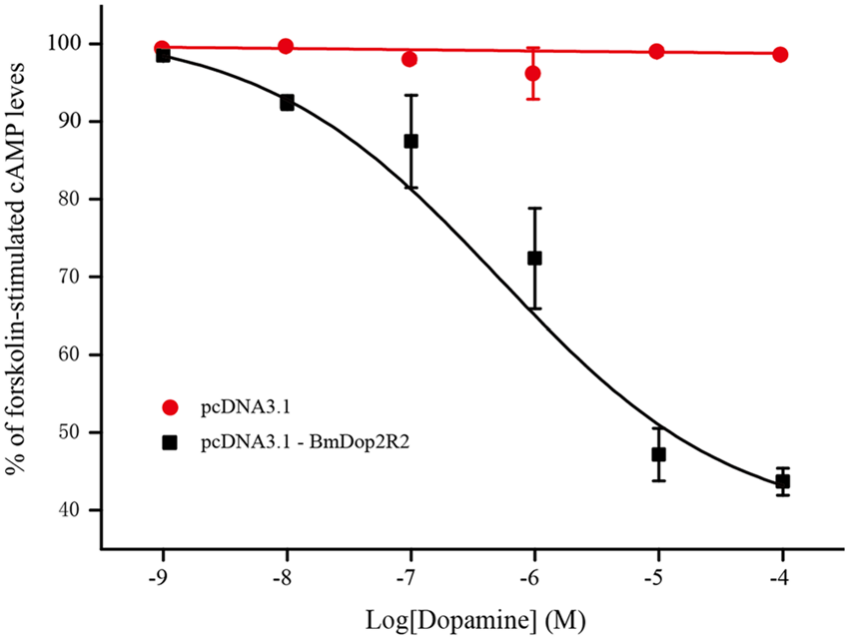
Dose-dependent effect of dopamine (1 nM to 100 μM) on intracellular cAMP levels in BmDop2R2-expressing cells exposing in 10 μM forskolin. The 100% level represents the cAMP amount in control cells treated with 10 μM forskolin. Each data point was measured in at least four or more independent assays. Error bars represent S.E.M.

For BmDop2R2 receptor, the dose-dependent effect of dopamine on cAMP levels was analyzed with concentrations ranging from 1 nM to 100 μM. The dopamine effect was concentration-dependent and saturable, resulting in a sigmoidal dose-response curve (Fig. 8). The intercellular cAMP levels was dramatically reduced at dopamine concentrations of ≥ 10^−8^M, and half-maximal activation (*EC*_*50*_) was achieved at a concentration of 6.57 × 10^−7^M (657 nM). When dopamine concentration was achieved at ≥ 10^−4^M, BmDop2R2 response was the maximum (Fig. 8). These results showed that dopamine activation of BmDop2R2 receptors was responsible for down regulation of cAMP levels.

## Discussion

Dopamine acts as a neurotransmitter, neuromodulator, and neurohormone in the nervous system, and depends on dopamine receptors to exert its effects in animals^4, 5^. In sponges, several genes corresponding to postsynaptic scaffolding have been reported^41^, but there is a lack in defined neuronal cell types^45^. This is consistent with the present study where no dopamine and other biogenic amine receptor genes are observed in the *A. queenslandica* genome. Homology search revealed that dopamine (biogenic amine) receptors appeared before the cnidarian divergence after the *A. queenslandica* split. Two distinct classes of dopamine receptors, D1-like and D2-like, are present in the phylogenetic tree and likely formed before the separation of deuterostomes and protostomes. The *C. elegans* Dop4 identified as D1-like receptor unique to invertebrates^22^ is located at the out-group of invertebrate Dop1R2 branch, suggesting that Dop1R2 probably as INDR split from its ancestor before the emergence of nematodes. Previous study has been revealed that there was only one D2-like gene in the genome of arthropod including Malacostraca (as *Panulirus interruptus* ^42^), Arachnida (as *Ixodes scapularis* ^43^) and Insecta (as *D. melanogaster* ^21^ and *A. mellifera* ^20^)^12^. However, we find two D2-like genes from the silkworm genome (Bombycidae) or other Lepidopteran genomes including plutellidae (as *P. xylostella*), pyralidae (as *A. transitella*) and papilionidae (as *P. machao*n). Two subclasses of Lepidoptera D2-like are clustered into a branch in insect D2-like group, suggesting that the similarity in protein is higher between Lepidoptera than among other species. Further, we find that two D2-like genes are closely linked on the same scaffold, and the chromosome region around D2-like gene loci show colinearity in Lepidopteran, but not in other species including Hmenoptera (as *A. mellifera*), Cleoptera (as *Tribolium castaneum*) and Dptera (as *D. melanogaster*). These indicate that two D2-like genes probably emerged by a gene duplication event taking place after Lepidoptera ancestor split from the common ancestor of Lepidoptera and Diptera. Furthermore, unequal crossing over usually generates tandem duplicated genes^46^, two D2-like genes loci in Lepidoptera are tandem arranged on one scaffold that possibly resulted from unequal crossing over.

Surprisingly, not only in Lepidoptera, there is also the vertebrates-specific collinearity related to D2-like receptor genes. Paralogous genes resulting from ancient duplications are often an indication of early syntenic regions, which was demonstrated for a large superfamily of homeobox genes^47^. Shared gene synteny is a reliable criterion to explore orthologs derived from genomic evolution. Thus, the synteny surround D2-like genes in this study is a strong reference for comparative genomics research in Lepidoptera and/or vertebrates.

Sequence analysis shows that BmDop2R2 has the complete characteristic structure of a GPCR protein, while BmDop2R1 is a truncated variant deleting N-terminus and TM I-II of GPCR protein. Interestingly, this deletion in BmDop2R1 is shared identity/similarity with that of predicted D2-like receptors from *P. xylostella* genome. These imply that the variant without regions of N-terminus to TM II are widely occured in Lepidoptera D2-like dopamine receptor. Currently, there are many reports on truncated GPCR having an absence of the sixth to seventh trans-membrane domains (TM VI-VII) and/or C-terminal region. For example, alternative splicing of the wild-type D2-like dopamine receptor DOP-3 resulted in the formation of DOP-3nf lacking TM VI-VII in *C. elegans* ^48^. Dop1R2B (a splice variant of INDR receptor Dop1R2A) has an alternative C-terminal region in *P. americana* ^19^. Here, truncated GPCR variant lacking N-terminus and trans-membrane1-2 domains (TM I-II) is the first report.

Truncated variants might associate with full-length receptors to modulate the signal transduction properties, which has been found in the D2-like receptor DOP-3 and its truncated splice variant DOP-3nf in *C. elegans* ^48^. DOP-3 attenuates forskolin-stimulated cAMP formation in response to dopamine, whereas DOP-3nf does not^48^. When DOP-3 was co-expressed with DOP-3nf, the ability to inhibit forskolin-stimulated cAMP formation was reduced^48^. Dop1R2B of *P. americana* is translated and transported to the plasma membrane but does not affect dopamine stimulation, and probably has a role to depress the capability of *P. americana* Dop1R2A ^19^. In this study, dopamine activating BmDop2R2-expressing cells decreases cAMP levels, whereas tyramine, octopamine, serotonin and histamine have no effect on intracellular cAMP. And the *EC*_*50*_ value of 6.57 × 10 M^−7^ and the maximal response value of ≥10^−4^M are similar to that of the *T. castaneum* Dop2 (D2-like) receptor (640 nM and 100 μM)^23^ for dopamine lowering cAMP levels, respectively. Thus, BmDop2R2 is a functional D2-like dopamine receptor. In contrast, dopamine stimulation does not affect cAMP levels for BmDop2R1. However, *BmDop2R1* as well as *BmDop2R2* are almost ubiquitously expressed in all tissues examined by RT-PCR, and encode protein having conserved sequence motifs and amino acid residues in the third (DRY), sixth (CWLP), and seventh (NPXXY) TMs (Fig. 2) that establish affinity and/or determine efficacy for endogenous amines^4^. Whether BmDop2R1 assembles and impairs BmDop2R2 function will be investigated in a forthcoming investigation and is beyond the scope of this study.

Gene duplication generates functional redundancy. Two genes with identical functions are unlikely to be stably maintained in the genome, but can be retained when they differ in some aspects of their functions^49^. Two D2-like receptors of Lepidoptera form two respectively independent branches, Dop2R1 branch and Dop2R2 branch, inside of Lepidoptera D2-like cluster, reflecting the proteins distinction which might be generated by the evolution of functional divergences for facilitating Lepidoptera-specific adaptation. Two duplicated genes via subfunctionalization interact to perform the original function that provides a mechanism for both duplicated gene copies being stably maintained in the genome^50^. *BmDop2R1* and *BmDop2R2* have difference in spatial expression pattern for each other. The cAMP levels are clearly down-regulated in HEK293 cells expressing BmDop2R2 but not BmDop2R1 for dopamine stimulation. It is possible that two D2-like genes are subfunctionalized to cooperate to carry out the function of parental gene in silkworm or other Lepidoptera during evolving.

## Conclusion

We identified and cloned two genes of D2-like receptors from the silkworm. This is different from a previous study in which only one D2-like receptor gene was found in insects and other arthropod. Our comparative genomics analyses suggested that the two genes are likely Lepidoptera-specific orthologs resulting from a gene duplication event in the common ancestor of Lepidoptera. We also found the truncated dopamine receptor deleting N-terminus to TM II region of GPCR structure for the first time, and demonstrated the function of D2-like receptor by expression cells. Our results provide some new insights into to understanding gene function of D2-like receptors in Lepidoptera and their evolutionary history.

## Methods

### Silkworm strains

Silkworm DaZao strains experimented were maintained at the Southwest University in china, and were reared under standard conditions.

### mRNA isolation and cDNA synthesis

Total RNA was purified using TRIzol reagent (Invitrogen) according to the manufacturer’s instructions, and cDNA was synthesized using oligo (dT) primers and Moloney murine leukemia virus (M-MLV) reverse transcriptase (Promega).

### Cloning

5’ RACE-ready cDNA was synthesized from the total RNA from the testis of the fifth silkworm using a GeneRacer^TM^kit (TaKaRa, Dalian, China) according to the provided manual (The sequence of all primers in Supplemental Table S2). The cDNA from the heads of adults were used as templates in PCR to amplify BmDop2R1 and BmDop2R2 (Supplementary Table S2). PCR reaction conditions included 94 °C for 3 min, 30 cycles of 94 °C for 30 s, 65 °C (BmDop2R1)/ 56 °C (BmDop2R2) for 1 min and 72 °C for 1.5 min and a final extension at 72 °C for 10 min. PCR products were isolated and cloned into the pMD19-Tsimple vector (Takara) and sequenced (as described in ref. 51).

### Semi-quantitative RT-PCR for expression analysis

Expression analysis by RT-PCR was performed using respective primers (Supplementary Table S2) for BmDop2R1 and BmDop2R2. Conditions of PCR consisted of 94 °C for 3 min, 30 cycles at 94 °C for 30 s, 55 °C for 40 s, 72 °C for 1 min and a final extension at 72 °C for 10 min. BmActin3 was used as an internal control. (as described in refs 51 and 52).

### Construction of expression vectors

The amplified products for the ORFs of BmDop2R1 and BmDop2R2 were ligated into the HindIII and NotI sites of the expression vector pcDNA3.1 (+) to produce the recombinant pcDNA3.1 (+)-BmDop2R1 and -BmDop2R2, respectively. Each insertion was confirmed by DNA sequencing.

### Transfections and cAMP assays

HEK-293 cells were grown in Dulbecco’s modified Eagle’s medium (D-MEM, Hyclone) supplemented with 10% fetal bovine serum (FBS, Invitrogen) at 37 °C with 5% CO_2_. Cells suspended in D-MEM containing 10% FBS were plated on 35-mm dishes 1 day before transfection. The attached cells were transfected with ~1.5 µg of the endotoxin-free plasmid in 350 µl of Opti-MEM I (Gibco) containng 3.5 µl Sinofection (Sino Biological). After incubation for 5 h at 37 °C with 5% CO_2_, the media was replaced with D-MEM containing 10% FBS (500 µl). And cell culture for 2 days, the culture media was removed and the drug diluted in Dulbecco’s modified Eagle’s medium was added into the stable transfected cells for incubation 30 min. The cells were washed two times with PBS (PH7.4) and lysed in 500 µl PBS by re-freezing before centrifugation at 2000 rpm for 20 min. The amount of intracellular cAMP extracted from harvested supernatant was determined using ELISA reagent (Huijia Biotech) according to the manufacturer’s instructions. Each measurement was performed in duplicate and a minimum of three independent assays were carried out for each compound and concentration tested.

### Multiple sequence alignment and phylogenetic analysis

Protein sequences similar to dopamine receptors were retrieved from GenBank (http://www.ncbi.nlm.nih.gov/genbank/). Multiple sequence alignments of the amino acid sequences were performed with DNAMAN and Clustalx. Transmembrane spanning domains were predicted by TMHMM (http://genome.cbs.dtu.dk/services/TMMM/; http://smart.embl-heidelberg.de/). Phosphorylation sites and N-glycosylation sites were predicted by NetPhos (http://www.cbs.dtu.dk/services/NetPhos/) and NetNGlyc (http://www.cbs.dtu.dk/services/NetNGlyc/) respectively. Values for identity (ID) and similarity (S) were calculated by BioEdit. We utilized MEGA 6.0 to calculate the genetic distances among different species and to construct neighbor-joining (NJ) trees with 1000-fold bootstrap resampling.

### Gene synteny

We examined the chromosomal localization of dopamine receptor genes and the neighboring genes in the available genomes of the NCBI (http://www.ncbi.nlm.nih.gov) Genome Browser.

### Species trees

Species trees in Fig. 5 was constructed using Cytochrome C protein whose accession numbers in GenBank were listed in Supplementary Table S1. Species divergence times, based on the assumption that *B.mori* and *D.melanogaster* diverged from 344.7 to 190.0 MYA^53, 54^, was estimated by the RelTime method.

## Electronic supplementary material

Supplementary Info File #1
