# Supplementary material for: Two dopamine D2-like receptor genes from the silkworm (Bombyx mori) and their evolutionary history in metazoan
Source: Sci Rep. 2017 Jul 28;7:6848. doi: 10.1038/s41598-017-07055-5 (PMC5533763; doi:10.1038/s41598-017-07055-5)
Supplement: Supplementary file 1 — Supplementary Info File #1 [file 41598_2017_7055_MOESM1_ESM.pdf]

Two dopamine D2-like receptor genes from the silkworm (*Bombyx mori*) and their evolutionary history in metazoan

Ping Chen, Peng Chen, Tian Li, Qi Shen, Deng-Feng Yan, Liang Zhang, Xi Chen, Yan Li, Wei Zhao

College of Biotechnology, Southwest University, Chongqing 400715, China

Corresponding authors:

Dr. Chen Ping

College of Biotechnology

Southwest University

2 Tiansheng Rd., Beibei District

Chongqing 400715 China

Tel: +86-23-68250084

Fax: +86-23-68250191

E-mail: chenping1918@swu.edu.cn

Table S1. The Accession number of the dopamine receptors in relevant species in this study.

Gene name	Species name	Accession number
MusD1	*Mus musculus*	BAC32050
MusD2	*Mus musculus*	BC105666
MusD3	*Mus musculus*	CAA47691
MusD4	*Mus musculus*	AAH16086
MusD5	*Mus musculus*	EDL37553
HomoD1	*human*	AAM18131
HomoD2	*human*	CAB56463
HomoD3	*human*	AAI28124
HomoD4	*human*	AAB59386
HomoD5	*human*	AAA52329
Homo GPR54	*human*	AAK83235
GallusD1	*Gallus gallus*	NP_001138320
GallusD2	*Gallus gallus*	BAP27884
GallusD3	*Gallus gallus*	ACR48171
GallusD4	*Gallus gallus*	BAP27927
GallusD5	*Gallus gallus*	XP_004936358
AlligatorD1	*Alligator sinensis*	XP_006028785
AlligatorD2	*Alligator sinensis*	XP_006026723
AlligatorD3	*Alligator sinensis*	XP_006020205
AlligatorD4	*Alligator sinensis*	XP_006019262
AlligatorD5	*Alligator sinensis*	XP_006016849
DanioD1	*Danio rerio*	ACI42369
DanioD2a	*Danio rerio*	AAN87174
DanioD2b	*Danio rerio*	AAP94011
DanioD2c	*Danio rerio*	AAP94012
DanioD3	*Danio rerio*	AAN87173
DanioD4a	*Danio rerio*	AAI29140
DanioD4b	*Danio rerio*	AAW80615
DanioD4c	*Danio rerio*	AAW80616
LampetraD2	*Lampetra fluviatilis*	ADO23655
Lampetra D1	*Lampetra fluviatilis*	AEW67331
PanulirusDop1R1	*Panulirus interruptus*	ABB87183
PanulirusDop1R2	*Panulirus interruptus*	ABB87182
PanulirusDop2	*Panulirus interruptus*	ABI64137
AplysiaDop	*Aplysia californica*	XP_005089278
AplysiaDop1R1	*Aplysia californica*	AAY00139
AplysiaDop1R2	*Aplysia californica*	XP_005099999
HydraDop	*Hydra vulgaris*	XP_012566961
SchistosomaDop-1	*Schistosoma mansoni*	CCD79389
SchistosomaDop-2	*Schistosoma mansoni*	CCD79386
SchistosomaDop2	*Schistosoma mansoni*	ACR77510

Gene name	Species name	Accession number
CaenorhabditisDop1	*Caenorhabditis elegans*	CCD68413
CaenorhabditisDop2	*Caenorhabditis elegans*	CAB03199
CaenorhabditisDop3	*Caenorhabditis elegans*	CCD83404
CaenorhabditisDop4	*Caenorhabditis elegans*	CCD65696
CaenorhabditisDop5	*Caenorhabditis elegans*	CAB07650
CaenorhabditisDop6	*Caenorhabditis elegans*	CCD65396
BombyxDop1R1	*Bombyx mori*	BAF98647
BombyxDop1R2	*Bombyx mori*	BAF98648
BombyxDop2R1	*Bombyx mori*	KY092434
BombyxDop2R2	*Bombyx mori*	KY092435
Bombyx FR	*Bombyx mori*	NP_001037007
Bombyx CytC	*Bombyx mori*	ACF41193.1
DrosophilaDop1R1	*Drosophila melanogaster*	AGB95944
DrosophilaDop1R2	*Drosophila melanogaster*	AAF56908
DrosophilaDop2(606)	*Drosophila melanogaster*	AAN15955
Drosophila CytC	*Drosophila melanogaster*	AAA28437
TriboliumDop1R1	*Tribolium castaneum*	DAA64500
TriboliumDop1R2	*Tribolium castaneum*	DAA64497
TriboliumDop2	*Tribolium castaneum*	DAA64499
ApisDop1R1	*Apis mellifera*	CAA73841
ApisDop1R2	*Apis mellifera*	AAM19330
ApisDop2	*Apis mellifera*	AY921573
Culex Dop2	*Culex quinquefasciatus*	EDS41903
AnophelesDop2	*Anopheles gambiae*	EGK97178
Aedes Dop2	*Aedes aegypti*	AEB73767
Musca Dop2	*Musca domestica*	XP_011291218.1
Ceratitis Dop2	*Ceratitis capitat*	XP_012161800.1
Plutella Dop1R1	*Plutella xylostella*	XP_011548761.1
Plutella Dop1R2	*Plutella xylostella*	XP_011548759.1
Plutella Dop2R1	*Plutella xylostella*	XP_011548761.1
Plutella Dop2R2	*Plutella xylostella*	XP_011548759.1
Plutella CytC	*Plutella xylostella*	AGN75105.1
Amyelois Dop1R1	*Amyelois transitella*	XP_013191513.1
Amyelois Dop1R2	*Amyelois transitella*	XP_013191520.1
Amyelois Dop2R1	*Amyelois transitella*	XP_013191513.1
Amyelois Dop2R2	*Amyelois transitella*	XP_013191520.1
Amyelois CytC	*Amyelois transitella*	XP_013199433
Papilio Dop1R1	*Papilio machaon*	XP_014357553.1
Papilio Dop1R2	*Papilio machaon*	XP_014357587.1
Papilio Dop2R1	*Papilio machaon*	XP_014357553.1
Papilio Dop2R2	*Papilio machaon*	XP_014357587.1
Papilio CytC	*Papilio machaon*	XP_014360070

Table S2. The primer sequences used in Materials and methods

		Cloning	Gene expression profiles	Quantitative RT-PCR
BmDop2R1	5’RACE Outer Primer	5'-CATGGCTACATGCTGACAGCCTA-3		
	5’RACE Inner Primer	5'-CGCGGATCCACAGCCTACTGATGATCAGTCGATG-3		
	5’RACE Specific Outer Primer	5'-GCATTCCCCGAGTCGTTGTTG -3		
	5’RACE Specific inner Primer	5'-GGACTCTTCCGCAACTGTCGCCAAC-3		
	F	5'-ATGTTTTGTCCACAGGTACA-3'	5'-AAGGCACTAAGAATACGAGCA-3'	5'- ATCTCCCTTTCTCCCCTGG-3'
	R	5'-CTTCAGTTCGGTAGCACGTAAA -3'	5'-CTAGCAAAAGAGGAAAACTCCT-3'	5'-TATTCCACAAGGCTCCACCAT-3'
BmDop2R2	F	5'-AAGAGAGACCACCGAACGC-3'	5'-AGAGCAAAAAAACAGAGGGTAG-3	5'-CGAGGAACTGGTCAACACGAG-3'
	R	5'-GGGCTAGGTGGTGCAATG-3	5'-CTATGGTGCCCAGCAGAAT-3'	5'-TGTGTTCGGGTCCACCATTG-3'
4A (sw22934)	F			5'-TTCGTACTGGCTCTTCTCGT-3’
	R			5'-CAAAGTTGATAGCAATTCCCT-3’

Figure S1

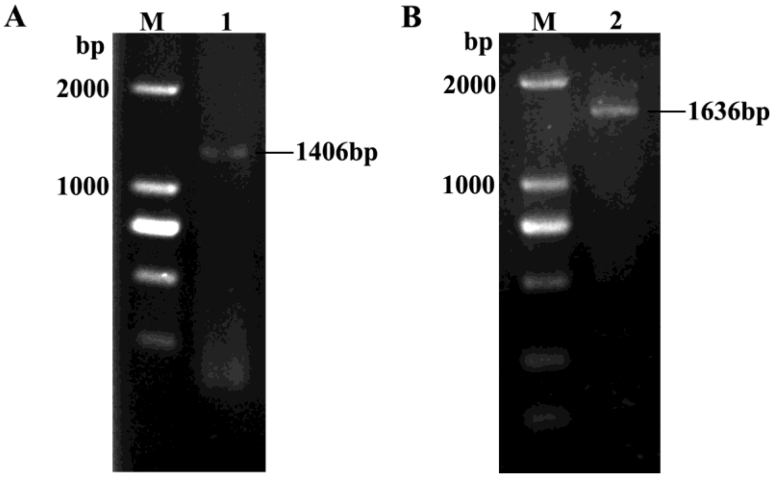

Figure S2

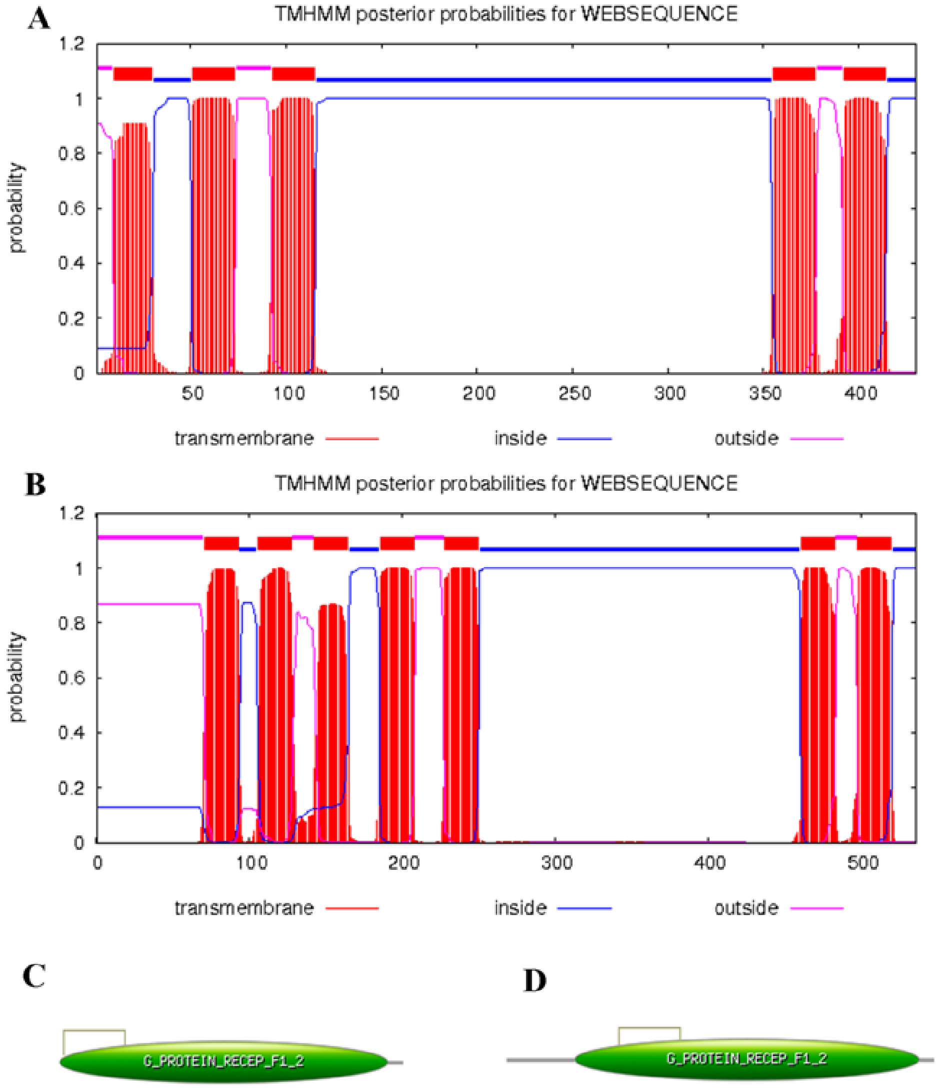

Figure S3

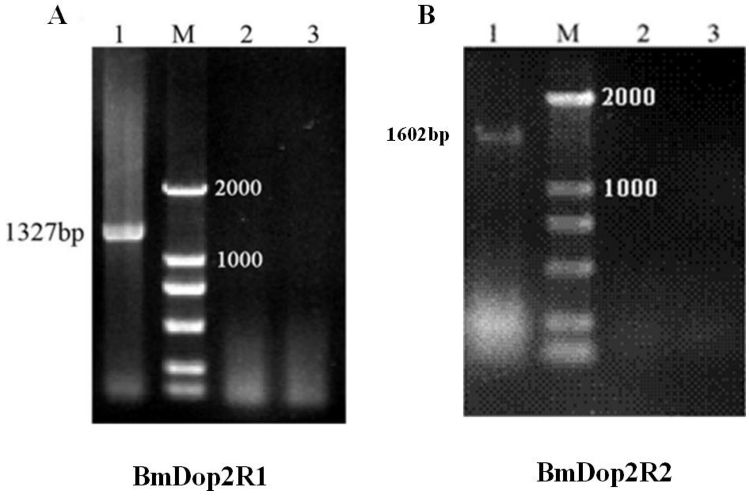

Figure S4

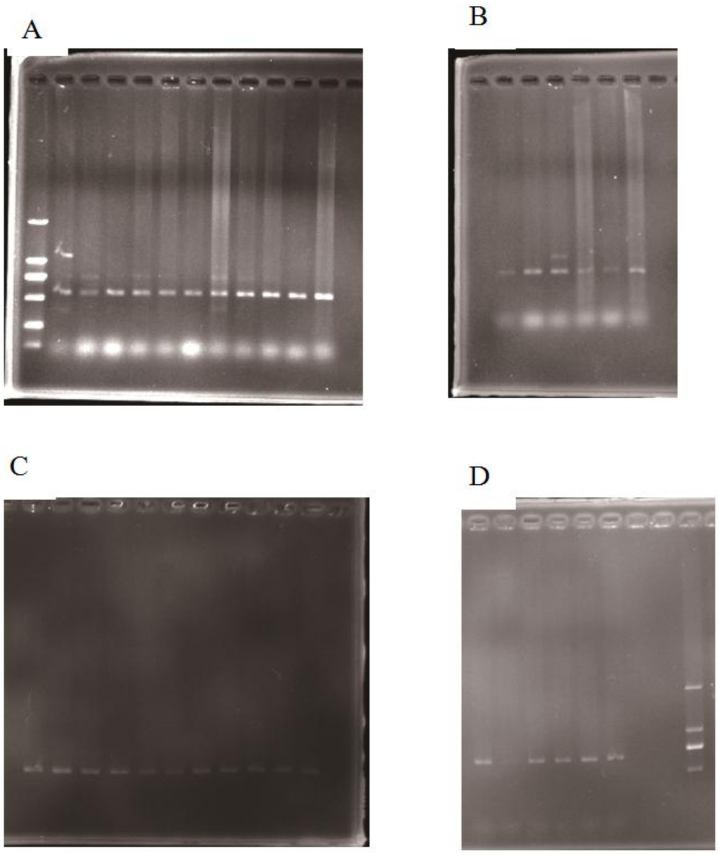

Titles and legends to figures (Supplementary Material)

Figure S1. PCR assay of the cloning plasmid of silkworm Dopamine D2-like receptor genes. Line indicates the target sequence. A - *BmDop2R1*. B - *BmDop2R2*.

Figure S2. Transmembrane region and domain of BmDop2R1 and BmDop2R2 by predicting on-line. A - Transmembrane region of BmDop2R1. B - Transmembrane region of BmDop2R2. C - domain of BmDop2R1. D - domain of BmDop2R2.

Figure S3. *BmDop2R1* or *BmDop2R2* expressing cells was analyzed by RT-PCR. A - *BmDop2R1*. B - *BmDop2R2*. Lane 1: pcDNA3.1-BmDop2R1/BmDop2R2; Lane 2: empty pcDNA3.1 vector; Lane 3: un-transfected

Figure S4. The uncropped and unprocessed original scans for all of the blots in Figure 6. A - BmDop2R1 in Figure 6 a1; B - BmDop2R1 in Figure 6 a2; C - BmDop2R2 in Figure 6 a1; D - BmDop2R2 in Figure 6 a2.
